# Maternal Milk Allopregnanolone May Buffer Negative Associations Between Maternal Postpartum Psychological Distress and Infant Regulatory Capacity

**DOI:** 10.1002/dev.70121

**Published:** 2026-01-28

**Authors:** Denise M. Werchan, Bradley Susskind, Rebecca Carpio, Brittany R. Howell, Natalie H. Brito, Moriah E. Thomason

**Affiliations:** ^1^ Department of Psychology UC Irvine Irvine California USA; ^2^ Department of Child & Adolescent Psychiatry NYU Langone New York New York USA; ^3^ Fralin Biomedical Research Institute at VTC Roanoke Virginia USA; ^4^ Department of Psychology Columbia University New York New York USA

## Abstract

Maternal psychological distress during pregnancy and the early postpartum period is a risk factor for dysregulated affective and regulatory function in young infants. Animal models suggest that perinatal stress may alter offspring development via allopregnanolone (ALLO) exposure. For example, variations in placentally derived ALLO in preterm infants have been linked with altered fetal neurodevelopment. However, no studies have investigated naturalistic variations in ALLO concentrations in maternal milk as a potential moderator of associations between maternal distress and infant temperament during the postnatal period. The current study assesses associations among ALLO concentrations in human milk, maternal psychological distress, and infant temperament in 81 mother‐infant dyads (31 females) measured at approximately 6.5 months postpartum (*M *= 6.55 months, range = 5.5–8 months). Results indicated that human milk ALLO concentration moderated effects of maternal psychological distress on infant regulatory capacity. Specifically, there was a negative association between maternal psychological distress and regulatory capacity in infants of mothers with below‐mean ALLO concentrations, but not in infants of mothers with above‐mean ALLO concentrations. However, there were no effects of ALLO on infant negative affect or surgency/positive affect. This study provides some of the first preliminary evidence that ALLO concentrations in human milk may moderate associations between maternal psychological distress and infant regulatory capacity.

## 1 Introduction

The perinatal environment is a robust modulator of child socioemotional and cognitive development (Monk and Fernández 2022). Substantial evidence demonstrates that maternal psychological distress, including symptoms of stress, depression, and anxiety, during the perinatal period is linked with negative developmental outcomes in offspring (Madigan et al. 2018). Prospective longitudinal studies with community‐based samples indicate that even subclinical symptoms of perinatal distress and mood dysregulation are associated with altered offspring neurodevelopmental outcomes (Fitzgerald et al. 2021; Meaney 2018). Given that over 30% of mothers are estimated to experience symptoms of psychological distress during the perinatal period (Meaney 2018), it is imperative to understand the modulators of these effects to support optimal maternal and child outcomes.

The development of infant temperament is thought to be particularly sensitive to effects of maternal psychological distress during the perinatal period. Temperament is defined as an individual's tendencies toward emotional arousal (both positive and negative) and the regulation of arousal through both volitional and automatic behaviors (e.g., attentional orienting and self‐soothing) (Derryberry and Rothbart 1997; Rothbart et al. 2000). Existing studies provide robust evidence that infants exposed to maternal psychological distress tend to exhibit more difficult temperamental traits such as irritability, fearfulness, and poorer self‐regulation (Feizabad et al. 2025; Lahti‐Pulkkinen et al. 2024; Bianco et al. 2023; Bush et al. 2017; Kingston et al. 2015). Importantly, these traits are predictive of negative long‐term outcomes in later life, including risk for externalizing and internalizing disorders and other forms of psychopathology (Morales et al. 2022). As such, understanding potential protective factors for infant temperament development is an important target for promoting optimal child outcomes.

Recent studies using animal models have identified allopregnanolone (ALLO) as a moderator of associations between maternal psychological distress and offspring neurodevelopment. ALLO is a naturally occurring neurosteroid synthesized from progesterone and is a robust modulator of GABA (gamma‐aminobutyric acid) signaling (Carver and Reddy 2013; Paul et al. 2020). Notably, maternal plasma and brain ALLO concentrations increase dramatically across human pregnancy, and low levels of ALLO have been implicated in the etiology of postpartum depression in birthing parents (Grötsch and Ehlert 2024; Brunton et al. 2014; Hellgren et al. 2014; Osborne et al. 2017). Placentally derived ALLO is also thought to be critical for fetal brain development, with recent studies indicating that the abrupt loss of placentally derived ALLO due to preterm birth is associated with altered neurodevelopment in human infants (Bakalar et al. 2022; Vacher et al. 2021).

Importantly, ALLO is responsive to both acute and chronic stress (Almeida et al. 2021; Henderson 2018; Bali and Jaggi 2014), which in turn may have protective effects on offspring neurodevelopment. For instance, exogenous ALLO administration has been shown to normalize offspring’ HPA axis reactivity in rodent models of maternal stress exposure (Brunton et al. 2015). Prenatal administration of ALLO to stressed dams during pregnancy also appears to ameliorate later anxiety‐like behavior in the offspring (Zimmerberg and Blaskey 1998). Similarly, a recent study found that postnatal administration of an ALLO analogue in guinea pigs appeared to reverse anxiety‐like behaviors induced by prenatal stress (Crombie et al. 2022).

However, the majority of prior studies have examined placental transmission of ALLO during prenatal development, and there are few studies examining potential effects of maternal ALLO concentrations during the postpartum period. This is an important gap in our knowledge, particularly since the first several months after birth are a sensitive period for establishing neural connections relevant to an infant's socioemotional and cognitive development (Knudsen 2004). Moreover, the early postpartum period (i.e., first 6 months) represents a time of increased susceptibility to stress and mood disorders in mothers (Shorey et al. 2018).

Recent pharmaceutical studies provide evidence that maternal ALLO may be detected in human milk. For instance, one study examining mothers receiving maternal brexanolone injections, a synthetic formulation of ALLO, found elevated concentrations of ALLO in milk, which closely mirrored maternal plasma concentrations (Wald et al. 2022). Similar findings are reported in a recent study examining milk composition in mothers receiving an oral formulation of ALLO to treat severe postpartum depression (Deligiannidis et al. 2024). Thus, human milk may provide an exogenous source of ALLO to infants during this period of rapid neurodevelopment in early postnatal life. However, no studies have investigated naturalistic variations in ALLO in human milk and possible links with maternal postpartum psychological distress or infant behavioral development.

To fill this gap, the current study assesses naturalistic variations in ALLO concentrations in maternal milk as a potential moderator of cross‐sectional associations between maternal psychological distress and infant temperament in a community‐based sample of mother‐infant dyads at approximately 6.5 months postpartum. Aligning with prior work, we expect that higher maternal psychological distress will be associated with more difficult infant temperaments (e.g., higher negative affect, lower regulatory capacity and surgency/positive affect). Importantly, however, we hypothesize that individual differences in ALLO concentrations in maternal milk will buffer associations between maternal psychological distress and infant temperament, decreasing the association between maternal psychological distress and difficult infant temperament.

## 2 Methods

### 2.1 Participants

Mothers were recruited from medical records as part of an ongoing, prospective longitudinal study in the New York City region. Mothers who provided milk samples at approximately 6.5 months postpartum were eligible for the current study, resulting in data from 81 mothers (*M* age = 33.5 years, SD = 4.0 years, range = 21.4–41.7 years) and infants (*n* = 31 females, *n* = 50 males; *M* age = 6.55 months, range = 5.5–8 months, SD = 0.67 months) tested between 12/2020 and 12/2021. All infants were born 36 weeks’ gestation or later (*M* gestational age = 39.1 weeks, SD = 1.4 weeks, range = 36–42 weeks) and were of normal weight at birth (*M* = 7.47 lb, SD = 1.2 lb, range = 5.4–12.3 lb). Full sociodemographic characteristics of the sample are reported in Table 1. The Institutional Review Board at NYU Langone approved all study protocols, and informed written consent was obtained electronically prior to study participation.

**TABLE 1:** Sample demographics.

	*N*	%
**Maternal education**		
<High school/GED	2	2.50
High school/GED	6	7.50
College degree	26	32.50
Graduate degree	46	57.50
**Annual household income**		
<$10,000	2	2.47
$10,000–$30,000	6	7.41
$30,000–$50,000	5	6.17
$50,000–$80,000	10	12.35
$80,000–$120,000	12	14.81
$120,000–$160,000	10	12.35
$160,000–$200,000	8	9.88
$200,000–$250,000	11	13.58
$250,000+	17	20.99
**Maternal race/ethnicity**		
White	46	57.50
Asian	7	8.75
Hispanic/Latine	14	17.50
Black/African American	5	6.25
Other	1	1.25
More than one race	5	6.25
Decline to answer	2	2.50

### 2.2 Measures

#### 2.2.1 Demographics

Information on maternal age, educational attainment, annual household income, and height and weight were collected. Maternal height and weight were used to compute body mass index (BMI).

#### 2.2.2 Maternal Psychological Distress

Self‐reported symptoms of perceived stress and postnatal depression/anxiety were used to generate a composite index of maternal psychological distress, as in prior work (Cohen et al. 2022; Madigan et al. 2024; Werchan et al. 2024). The perceived stress scale (PSS; Cohen et al. 1983) is a widely used 14‐question survey designed to capture the degree to which the respondent has perceived situations as stressful within the last month. Items are rated on a five‐point Likert scale ranging from 0 (never) to 4 (very often). A total score is the sum of scores from all items (after four items are reverse scored). Scores can range from 0 to 40. This scale had a Cronbach's alpha of 0.90, indicating excellent reliability. The Edinburgh postnatal depression scale (EPDS; Cox et al. 1987) is a 10‐item self‐report scale assessing common symptoms of depression and anxiety during the perinatal period. Items are rated on a five‐point Likert scale ranging from 0 to 4, and a total postpartum depression score is calculated by summing all items (after two are reverse scored). This scale had a Cronbach's alpha of 0.87, indicating good reliability.

Summary scores on the EPDS and PSS were highly correlated, *r*(76) = 0.76, *p *< 0.001, and were combined to form a single composite index of maternal psychological distress. Specifically, the total EPDS and PSS summary scores were first standardized, and then the standardized values were averaged to generate an overall maternal psychological distress score. This approach ensures that each measure is weighted equally and that the composite score represents overall deviation from the mean for each subject.

#### 2.2.3 Infant Temperament

Infant negative affect, surgency/positive affect, and regulatory capacity were assessed using the infant behavior questionnaire—very short form (IBQ‐VSF; Gartstein and Rothbart 2003). The IBQ‐VSF is a condensed, 37‐item parental report measure of infant temperament. Mothers rate the prevalence of a number of behaviors related to infant negative affect, surgency/positive affect, and regulatory capacity on a seven‐point Likert scale (1—*never*, 2—*very rarely*, 3—*less than half the time*, 4—*about half the time*, and 5—*more than half the time*, 6—*almost always*, 7—*always*). IBQ‐R VSF scores are shown to have strong internal consistency and test‐retest reliability (Putnam et al. 2014). Cronbach's alpha in our sample was 0.86 for negative affect, 0.81 for surgency/negative affect, and 0.64 for regulatory capacity, indicating adequate to good reliability.

#### 2.2.4 ALLO Milk Concentrations

Participants who consented to provide adult milk samples received a package containing a new Haakaa silicone manual breast pump, a sterile tube with ∼60 mL capacity, a biohazard bag for storing the collected sample, two frozen gel packs, an insulated box, collection instructions, and a FedEx Express return label. Before collection, participants were instructed to wash their hands and clean the collection site (nipple, areola, and surrounding skin) with soap and water and then allow the area to dry completely. They were then asked to collect 40 mL of milk, using either the provided breast pump or by hand expression. After collecting the sample, participants were asked to securely seal the tube, place it in the biohazard bag, and immediately place it on ice for return shipment that same day. Participants also completed a collection questionnaire (available open‐source at https://doi.org/10.17605/OSF.IO/UQHCV), which included information regarding the time the milk sample was collected.

Upon receipt, the samples were vortexed for 25 s, aliquoted into smaller volumes (1, 5, and 10 mL), and placed in a −80°C freezer for long‐term storage. A volume of 6 mL for each milk sample was shipped to collaborators overnight on dry ice for analyses. Upon collaborator's receipt, these samples were immediately placed in a −80°C freezer ensuring samples were not thawed prior to assaying. Subsequently, 1 mL was thawed for ALLO measurement, and the thawed milk was gently centrifuged (880 g for 10 min) and manually defatted. Abcam's ALLO ELISA kit (ab284029) was used for assays, which has intra‐ and inter‐assay coefficients of variation (CVs) of 10.5 and 6.5, respectively. It has been validated for use with human milk and demonstrates low cross‐reactivity (≤0.3%) with a broad panel of related steroids, including progesterone, testosterone, cortisol, and corticosterone. We used 25 µL of sample per assay in duplicate, per the manufacturer's instructions. ALLO levels were determined in pg/mL. To ensure the reliability of measurements, each plate included a full standard curve and a negative control, run in duplicate or triplicate. All standard curves were consistent with expected results provided by the manufacturer. We verified that there was no correlation between ALLO concentrations and the time of day that milk samples were collected, *r *= −0.14, *p *= 0.27.

### 2.3 Analytic Plan

All analyses were performed using the Lavaan package in R version 4.0.2. We first examined descriptive statistics and bivariate correlations among maternal psychological distress, ALLO, and infant outcomes. Possible maternal (i.e., annual household income, caregiver age, caregiver educational attainment, and caregiver BMI) and infant (i.e., age at data collection, gestational age at birth, biological sex, and birthweight) variables that might influence predictors or outcomes were evaluated using correlations and *t* tests. Variables associated with maternal psychological distress, ALLO concentrations, or infant outcomes at the level of *p* < 0.10 were flagged for inclusion as covariates in all analyses. Infant age and household income met this criterion and were thus included as covariates in all analyses.

We used multiple linear regressions to examine effects of maternal psychological distress, ALLO concentrations, and their interaction in predicting variability in infant temperament. We included the interaction between maternal psychological distress and ALLO concentrations to test our primary hypothesis that ALLO may buffer the effects of maternal psychological distress on infant temperament. All independent variables were mean centered prior to computing the interaction term. Separate models were run for infant negative affect, surgency/positive affect, and regulatory capacity. Subsequent exploratory analyses were conducted to assess potential differential effects in mothers who were exclusively breastfeeding versus those who were not.

To account for missing data within the sample (2% on the predictor variable, 18.5% on the moderator, and 1% on the outcome variable) and to prevent bias in model estimation due to missing data, we used full‐information maximum likelihood estimation (FIML). Relative to other methods (e.g., listwise or pairwise deletion, mean imputation), FIML generates unbiased estimates for data missing at random and is considered a superior method for handling missing data. Little's Missing completely at random (MCAR) test showed that the data fit an MCAR pattern, *χ* ^2^ = 107.87, *p* = 0.16, and is thus appropriate to model using FIML. In addition, we also used direct comparisons to verify that there were no differences in any infant or maternal characteristics between mothers with versus without viable milk samples, *p*_s_ > 0.11, or between mothers who reported exclusively breastfeeding versus those who did not, *p*_s_ > 0.46.

## 3 Results

Means, standard deviations, and correlations among all study variables and covariates are presented in Table 2.

**TABLE 2:** Descriptive statistics and correlations among all study variables and covariates.

	Variable	*M*	SD	1	2	3	4	5	6	7
1	ALLO concentration (pg/mL)	29.7	12.57	1						
2	Maternal psychological distress	−0.01	0.94	0.12	1					
3	Infant negative affect	4.07	1.12	0.05	0.21	1				
4	Infant regulatory capacity	5.40	0.60	0.13	−0.17	0.14	1			
5	Infant positive affect/surgency	5.40	0.60	0.08	−0.19	0.41^**^	0.27^*^	1		
6	Infant age	6.55	0.67	0.17	−0.08	0.12	−0.07	0.42^***^	1	
7	Household income^a^	6.0	2.37	0.02	−0.14	−0.33^**^	−0.13	−0.26^*^	−0.18	1

Abbreviation: ALLO, allopregnanolone.

^a^ Income was coded as 1 = <10k, 2 = 10–30k, 3 = 30–50k, 4 = 50–80k, 5 = 80–120k, 6 = 120–160k, 7 = 160–200k, 8 = 200–250k, 9 = 250k+.

* *p *< 0.05.

** *p *< 0.01.

*** *p *< 0.001.

**FIGURE 1:**
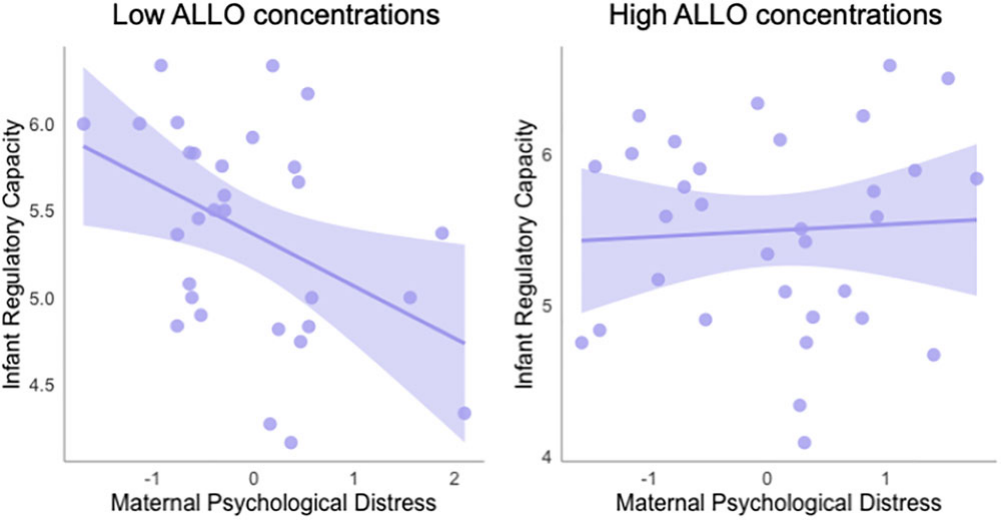
Higher maternal psychological distress predicted lower infant regulatory capacity in mothers with below‐median levels of ALLO (left panel); however, this association was ameliorated in mothers with above‐median levels of ALLO. ALLO, allopregnanolone.

We used regression analyses to examine the contribution of maternal psychological distress and ALLO, as well as their interaction, in predicting infant temperament. All models controlled for household income and infant age. As shown in Table 3, there were significant direct effects of maternal psychological distress on both infant regulatory capacity and negative affect. In addition, significant effects were found for the interaction of maternal psychological distress and ALLO on infant regulatory capacity. Figure 1 depicts the interaction of maternal psychological distress and ALLO, which shows that negative associations between maternal psychological distress and infant regulatory capacity were ameliorated in infants of mothers with higher concentrations of ALLO detected in their milk samples.

**TABLE 3:** Full regression results.

	Regulatory capacity		Negative affect			Positive affect/surgency	
Predictor variable	*B* (SE)	*β*	*B* (SE)		*β*	*B* (SE)	*β*
Maternal psychological distress	−0.14 (0.07)	−0.21^**^	0.20 (0.13)	0.17^*^		−0.21 (0.10)	−0.21^**^
ALLO concentration	0.01 (0.01)	0.10	0.00 (0.01)	0.02		0.01 (0.01)	0.06
Maternal distress × ALLO	0.02 (0.01)	0.27^**^	0.01 (0.01)	0.05		0.00 (0.01)	−0.02
Household income	−0.06 (0.03)	−0.23^**^	−0.15 (0.05)	−0.30^***^		−0.09 (0.04)	−0.23^**^
Infant age	−0.20 (0.10)	−0.22^*^	0.08 (0.20)	0.05		0.50 (0.15)	0.37^***^

Abbreviation: ALLO, allopregnanolone.

*p < 0.10.

**p < 0.05.

***p < 0.01.

Finally, we conducted exploratory multiple linear regression analyses to examine these effects separately in mothers who reported exclusively breastfeeding (*N* = 54) relative to those who were not exclusively breastfeeding (*N* = 27). Infant age and household income were again included as covariates. In mothers who reported that they were not exclusively breastfeeding their infants, the interaction between maternal psychological distress and ALLO was no longer significantly associated with infant regulatory capacity, *β* = 0.31, *p* = 0.24; however, in mothers who were exclusively breastfeeding, this interaction remained significant, *β* = 0.37, *p* = 0.006.

## 4 Discussion

Temperament modulates an infant's sensitivity to their environment, thereby setting the foundation for subsequent socioemotional and cognitive development. Here, we found that ALLO concentrations in human milk moderated associations between maternal postpartum psychological distress and infant regulatory capacity at approximately 6.5 months of age. This study provides the first preliminary evidence in human infants that ALLO may help buffer adverse effects of maternal psychological distress on infant outcomes during the postnatal period.

Infant regulatory capacity is an important aspect of temperament involved in regulating arousal and thus plays a substantial role in learning, emotion, and the development of more sophisticated self‐regulation abilities. Accordingly, prior studies have linked variation in infant regulation as an important risk and protective factor for maladaptive outcomes in later life, including psychopathology (Morales et al. 2022; Rothbart and Posner 2015). Replicating prior work, we observed that maternal psychological distress was correlated with higher infant negative affect and lower regulatory capacity traits. Importantly, however, we also found that maternal ALLO concentrations appeared to buffer adverse effects of maternal psychological distress on infant regulatory capacity. This observation is consistent with the hypothesized role of ALLO in moderating reactivity of the HPA axis (Bali and Jaggi 2014). In turn, this may protect against the adverse effects of stress exposure on the development of neural regions critical for behavioral and emotional regulation (Blair and Raver 2012).

This study adds to growing evidence indicating an important role for ALLO in regulating brain and cognitive development more generally. Indeed, ALLO plays a key role in many neurodevelopmental processes, including neurogenesis, cell survival, synapse stabilization, and myelination (Schumacher et al. 2014). Consistent with these findings, studies also indicate that ALLO may have neuroprotective effects in the fetal brain in response to pregnancy and childbirth complications (Brunton et al. 2014; Pluchino et al. 2016). Placental ALLO insufficiency has also been linked with cerebellar myelination abnormalities and autistic‐like behaviors in offspring (Vacher et al. 2021). Of note, however, the majority of existing knowledge is limited to the prenatal period. This is the first investigation of maternal ALLO concentrations during the postnatal period. Moreover, this study also reports the first evidence of associations between maternal ALLO concentrations and early cognitive and socioemotional outcomes in human infants.

In addition, this is the first study to our knowledge examining potential oral ingestion of ALLO to infants. Generally, ALLO has low oral bioavailability (Schüle et al. 2014), and pharmaceutical administrations of synthetic analogues of ALLO typically rely on intravenous infusion (Marecki et al. 2023). However, recent pharmaceutical advances have found that binding an oral, synthetic analogue of ALLO to lipids increases its oral bioavailability by allowing the drug to bypass the liver and instead move directly from the gut into lymphatic vessels that process dietary fats (Althaus et al. 2020; Marecki et al. 2023). Given the complexity of lipids and milk fat globules and evidence for progesterone in their regulation (Argov‐Argaman et al. 2020), it is possible that the lipid composition of maternal milk could increase the oral bioavailability of ALLO and thus ingestion by infants. More likely, however, is that the observed moderating effects of ALLO may co‐occur with other biological and/or behavioral mechanisms that may have independent or complementary impacts on infant development. For example, ALLO concentrations may covary with other components of milk that also have known impacts on infant brain and cognitive development, such as maternal cortisol levels or the fat composition of milk (Blair and Raver 2012; Innis 2007). It is also plausible that our findings could reflect shared genetic variance in genes regulating GABAergic signaling, given known associations between GABA and both ALLO (Diviccaro et al. 2022) and temperament (Gonda et al. 2021; Zwir et al. 2020). An additional possibility is that postnatal ALLO levels may covary with prenatal ALLO levels, where placental transmission may directly impact fetal brain development (Pluchino et al. 2016). These are active areas for future research.

As such, the current findings cannot speak to the mechanisms or route of transmission underlying these effects. However, to better discern oral ingestion as a candidate pathway to explore in future work, we conducted exploratory analyses examining these effects separately in mothers who were exclusively breastfeeding and in mothers who were not. If these results relate to oral ingestion of ALLO, we would expect stronger effects in infants with greater intake of milk. Interestingly, our exploratory analyses were consistent with this hypothesis. Specifically, we observed that the interaction among maternal psychological distress and ALLO on infant regulatory capacity was stronger in the exclusively breastfeeding mothers, whereas it was non‐significant in those who were not exclusively breastfeeding. This finding provides support for the possibility that oral transmission of ALLO or other covarying components of milk may contribute to the observed effects. However, these results should be interpreted cautiously, particularly as we had a relatively small sample and only a coarse measure of breastfeeding exposure. Moreover, exclusive breastfeeding co‐occurs with other factors that may independently support infant development, such as increased physical closeness, maternal sensitivity, and infant attachment security (Linde et al. 2020; Peñacoba and Catala 2019). Likewise, maternal psychological distress and infant temperament can also influence breastfeeding patterns (Nagel et al. 2022; Vilar‐Compte et al. 2022), raising the possibility of potential self‐selecting or bidirectional effects. As such, future work should use more detailed assessments of breastfeeding frequency and quality, along with broader behavioral and contextual measures, to better disentangle these complex pathways.

Interestingly, our results indicated that ALLO did not moderate effects of maternal psychological distress on infant positive affect/surgency or negative affect. Surgency and negative affect reflect an individual's tendencies toward positive and negative emotional arousal, which are thought to decrease and increase susceptibility to mood disorders, respectively (Dougherty et al. 2010; Eisenberg et al. 2009; Morales et al. 2022). Given that ALLO also has a recognized role in mood disorders (Chen et al. 2021), these null findings are somewhat surprising. However, we found that ALLO moderated effects of maternal psychological distress on infant regulatory capacity, which reflects temperamental differences in the ability to regulate emotional arousal and in the recovery from distress (Derryberry and Rothbart 1997; Rothbart et al. 2000). The regulation of emotional arousal is thought to be a robust protective factor for preventing future mood dysregulation (Rothbart and Posner 2015). As such, our findings are consistent with the recognized role of both ALLO and infant regulatory capacity in risk for mood and anxiety disorders.

The specificity of the observed effects of ALLO in moderating infant regulatory capacity is also consistent with ALLO's role as a GABA‐ergic modulator. Indeed, GABA has been shown to regulate attention and working memory (Koh et al. 2023). Moreover, reduced GABA concentrations are often seen in both mood disorders (Cutler et al. 2023) and in neurodevelopmental disorders associated with impaired self‐regulation, such as ADHD (Edden et al. 2012; Puts et al. 2020). For example, previous findings have shown that serum ALLO levels are significantly lower in children with ADHD relative to healthy controls (Şahin et al. 2022). Similar findings in rodent models have shown that neonatal ALLO interacts with prenatal stress to predict impulsivity and novelty‐seeking behaviors in later life (Bartolomé et al. 2020).

There are several limitations of this work. First, we tested a relatively small sample of infants in a highly educated, community‐based sample. We also lacked detailed measures of maternal breastfeeding practices or medication exposure, which are important factors to consider in future work. In addition, we also only measured concurrent associations among maternal psychological distress, ALLO, and infant outcomes at approximately 6.5 months postpartum. Thus, we cannot discern whether these findings are enduring or transient. Future longitudinal investigations are important for understanding whether maternal ALLO concentrations in the postnatal period are associated with altered trajectories of development. In addition, longitudinal investigations in larger samples will also be imperative for better inferring potential mechanisms from human data. This is particularly important with respect to the translation of basic science research to inform clinical decision‐making.

In summary, this study provides the first investigation of postnatal maternal ALLO concentrations in human milk and its associations with maternal postpartum psychological distress and infant outcomes. Importantly, we provide the first preliminary evidence in human infants that ALLO may help buffer negative associations between maternal psychological distress and infant regulation. This work sheds novel light into the biomarkers and possible mechanisms underlying associations among maternal psychological distress and infant development. Replicating these findings in future samples will be essential for increasing our knowledge and promoting optimal maternal and child outcomes.

## Funding

This work was funded by NIH R01MH125870 and the NYU COVID Catalyst grant (to NHB), NIH R01MH126468 (to MET), and by a Brain and Behavior Research Foundation NARSAD Young Investigator Award and an SRCD Early Career Award (to DMW).

## Conflicts of Interest

The authors declare no conflicts of interest.

## Data Availability

The data analyzed in the current study are available from the corresponding author on reasonable request.
